# Translation and psychometric evaluation of the Korean version of the Reproductive Concerns After Cancer Scale (RCAC)

**DOI:** 10.1186/s12912-024-02171-w

**Published:** 2024-07-29

**Authors:** Sook Jung Kang, Hae Jeong An, Woon Young Hwang, Hyerim Lee, Yoonjung Kim

**Affiliations:** 1 College of Nursing, Ewha Womans University, #203-2 Hellen Hall, 52, Ewhayeodae-Gil, Seodaemun-Gu, Seoul, 03760 Republic of Korea; 2 College of Nursing, Konyang University Medical Campus, #209, 158, Gwanjeodong-Ro, Seo-Gu, Daejeon, 35365 Korea

**Keywords:** Cancer survivors, Women, Reproduction, Fertility, Oncology nursing, Nursing

## Abstract

**Background:**

Improving cancer survival rates highlights post-treatment fertility implications for reproductive-aged women. To provide fertility care for cancer survivors, nurses need instruments to assess and communicate reproductive concerns with cancer survivors and healthcare providers. This study aimed to translate the Reproductive Concerns after Cancer Scale (RCAC) into Korean and examine its psychometric properties in young female cancer survivors in South Korea.

**Methods:**

The RCAC was translated into Korean, involving expert bilingual translators for initial translation and reverse translation for cultural and semantic accuracy. In detail, the RCAC was translated into Korean and evaluated in a preliminary study involving 10 cancer survivors. Subsequently, a revised version of the instrument was administered to 182 cancer survivors and a psychometric evaluation was conducted. The process included verifying content validity, and then assessing construct validity using exploratory factor analysis and criterion validity. The reliability of the instrument was quantified by measuring its internal consistency using Cronbach’s alpha.

**Results:**

The translated RCAC demonstrated an item-level content validity index of 1.0 and a scale-level index of 1.0. The content was finalized based on preliminary survey findings, which revealed that all participants thought the instrument was clear. The Korean version of the RCAC demonstrated a satisfactory level of validity per exploratory factor analysis, which resulted in a 14-item instrument consisting of three subscales: “fertility potential” (six items), “health problem” (five items), and “acceptance” (three items). The items and subscales explained 57.6% of the variance. Criterion validity was confirmed through an analysis of the correlation between the Korean version of the RCAC and both the FACT-G (*r* = -0.36, *p* < .001) and PHQ-9 (*r* = 0.38, *p* < .001). Cronbach’s alpha coefficient of the Korean version of the RCAC was 0.83.

**Conclusions:**

The Korean version of the RCAC is a valid and reliable instrument for assessing reproductive concerns in female cancer survivors. Thus, this instrument can be used to provide tailored care to female cancer survivors of reproductive age by clarifying and assess their reproductive concerns. This may support the development of guidelines or policies to provide care for those with reproductive concerns who require nursing care.

## Background

Owing to recent advancements in healthcare systems, cancer survival rates have increased. The 5-year cancer survival rate is 93.6% and is steadily increasing [1]. In women of reproductive age, better survival raises concerns regarding fertility after successful cancer treatment, resulting in higher levels of distress, depressive symptoms, and a poorer quality of life [2–4]. Fertility problems can harm marital and other social relations [4, 5]. Therefore, addressing fertility issues should be a high priority for clinicians during and after cancer treatment. However, some cancer survivors may feel that their concerns are not sufficiently addressed by providers [6].

South Korea has more than twice as many young women with breast cancer under the age of 45 as the United States [7, 8], and the number is steadily increasing. Meanwhile, South Korea has an average fertility age of 33.5, which is the highest among the Organization for Economic Co-operation and Development (OECD) countries and is steadily rising [9]; the fertility rate over 30 has also risen [10]. Given the increase in younger breast cancer survivors and the higher fertility age, fertility is a critical issue for women who have never been pregnant and have experienced childbearing before their cancer diagnosis. Therefore, healthcare providers should consider cancer survivors’ concerns about fertility; however, there are limitations to reproductivity after cancer [11].

A valid and reliable screening instrument may help cancer survivors discuss fertility concerns with their medical teams. Tools related to fertility measure the patients’ knowledge about fertility [12] and fertility intentions [13]. Instruments that measure fertility concerns in patients with cancer include the Reproductive Concerns Scale [14] and the Reproductive Concerns After Cancer (RCAC) [6, 15]. The Reproductive Concerns Scale is a 14-item instrument that focuses on feelings and thoughts related to fertility and pregnancy [14]. On the other hand, the RCAC, with 18 items, takes a more comprehensive approach to reproductive concerns by measuring concerns related to the family and the cancer survivor, such as children’s health and partner disclosure, while including questions about the acceptance of possible infertility. In Korea’s cultural norms, family needs are more important than individual requirements [16]. Therefore, reproductive issues related to cancer in South Korea should be considered by families and cancer survivors. Therefore, RCAC is appropriate for a better understanding of fertility concerns in South Korea.

The original Reproductive Concerns After Cancer (RCAC) scale was developed to measure fertility and childbearing concerns among cancer survivors [6, 15]. This instrument has shown satisfactory construct validity and internal consistency among cancer survivors in the United States. Moreover, RCAC has been used in various countries, including China [17], Sweden [2], and Portugal [18]. However, the factor structure of an instrument often differs from the original because of its cultural context. The Chinese and Swedish versions had a personal health subscale [2, 17]. On the other hand, in the Portuguese version of the instrument, the items in the personal health subscale were divided into children's health risk, barriers to getting pregnant, and fertility potential [18]. In South Korea, strong bonds and family stability are valued more highly than individual rights [19]. Moreover, Korean women tend to think of their children as their alter egos [19, 20]. This suggests that Korean women’s ‘personal health’ may include their children as well as themselves. Also, this family-centered culture may influence Korean women’s reproductive concerns. When translating and revising RCAC, due to this variety, there is a need to factor in cultural influence regarding motherhood as well as perception of personal health.

In addition, it is necessary to identify the applicability of RCAC in Korea considering the pregnancy age of Koreans. China [17], Sweden [2], and Portugal versions [18] of RCAC are targeting young breast cancer survivors under the age of 40. In Korea, the number of women over 40 giving birth has more than doubled in the past decade, accounting for 8.2% of all births, and continues to rise [21]. Therefore, it is necessary to evaluate the psychometric properties of the RCAC in Korean breast cancer survivors, including those in their 40s.

To ensure proper oncofertility care, it is important to identify and communicate the factors that contribute to reproductive concerns among cancer survivors. Particularly, nurses are responsible for providing personalized care to address the reproductive concerns of cancer survivors. Therefore, nurses should use appropriate assessment tools to evaluate reproductive concerns in female cancer survivors, which is essential to identify and assess the reproductive issues faced by young cancer survivors in Korea. This study aimed to translate the RCAC scale into Korean considering cultural aspects and confirm the reliability and validity of the Korean version of the RCAC.

## Methods

### Study design

This was a psychometric study to examine the reliability and validity of the Korean version of the RCAC developed by JR Gorman, HI Su, JP Pierce, SC Roberts, SA Dominick, and VL Malcarne [6] to measure the reproductive concerns of cancer survivors.

### Study participants

The participants were female cancer survivors. The specific inclusion criteria were as follows: 1) women diagnosed with cancer aged between 20 and 49 years; 2) those who had completed active cancer treatment (for breast cancer, women who were taking hormone therapy, such as tamoxifen, were included); and 3) native Korean speakers. Participants were excluded if they had reached menopause, were unable to become pregnant, or did not meet the study’s inclusion criteria.

The number of participants was determined by considering that factor analysis requires at least ten times the number of items in the original instrument [22]. As the original instrument consisted of 18 items, we recruited 228 participants, anticipating a 20% dropout rate. Online surveys tend to show a response rate approximately 10% lower than that of offline surveys [23]; therefore, in this study, a 20% dropout rate was accounted for. Forty-six women who did not complete the survey, indicating that they did not want to participate, were excluded from the study. Based on the survey questions, the participants were informed that they should not complete the survey if they did not want to participate. A total of 182 cancer survivors completed the survey, and their data were used to meet the criterion of a sample size of at least 10 times the number of original instrument items [22]. Given the original 18 items of the instrument, a sample size greater than 180 was considered appropriate.

### Instruments

Referring to previous research showing that reproductive concerns are associated with poor QoL and increased depressive symptoms [2–4, 17], instruments for both QoL and depressive symptoms were used in this study. The same instruments for both quality of life and depressive symptoms used in the original instrument [6] were used in this study.

#### The Reproductive Concerns After Cancer Scale (RCAC)

The original RCAC was developed for female cancer survivors by Gorman et al. [6] and includes 18 items in six subcategories (fertility potential, partner disclosure, child’s health, personal health, acceptance of possible infertility, and becoming pregnant). Participants responded using a five-point Likert-type scale ranging from 1 (strongly disagree) to 5 (strongly agree), with higher mean scores indicating a higher level of reproductive concern (possible range: 1–5). Cronbach’s alpha was 0.82 in Gorman et al. [6], indicating good or acceptable reliability for the six sub-scales (α = 0.78–0.91).

#### Functional Assessment of the Cancer Therapy-General Scale (FACT-G)

To measure the participants’ quality of life, the Korean version of the Functional Assessment of Chronic Illness Therapy-General (FACT-G) scale [24] was used. The FACT-G consists of 27 items in four subcategories (physical, social/family, emotional, and functional well-being). Participants responded using a five-point Likert scale ranging from 0 (not at all) to 4 (very much), with higher overall summation scores indicating a higher level of quality of life (possible range: 0–108). Cronbach’s alpha was 0.87 in the Korean version [24] and 0.88 in this study.

#### Patient Health Questionnaire-9 (PHQ-9)

The Korean version of the Patient Health Questionnaire-9 (PHQ-9) to measure participants’ depressive symptoms [25]. The PHQ-9 includes nine items on the frequency of depressive symptoms in the past two weeks. Participants responded using a four-point Likert scale ranging from 0 (never) to 3 (every day), with higher overall summation scores indicating severe depression (possible range: 0–27). The instrument’s Cronbach’s alpha was 0.87 in the Korean version [25] and 0.87 in this study.

### Process

#### Translation

Prior to translation, permission was obtained from the authors of the original instrument for translation and psychometric evaluation. The original RCAC scale was translated into Korean and culturally adapted to measure reproductive concerns using the World Health Organization guidelines for instrument translation [26]. Two bilingual translators independently translated the original RCAC from English into Korean. The first translator understood nursing care and the second understood cancer survivor care. The translators possessed clinical experience as nurses in both the United States and South Korea and were fluent in both English and Korean. One was a graduate of a nursing school in South Korea, while the other held a doctoral degree in nursing from the United States. The authors deliberated on the translation by considering wording, vocabulary, and clarity and determined whether cultural adjustments were necessary. Subsequently, a reverse translation into English was conducted by two bilingual translators blinded to the original version. Of the two translators who were not involved in the initial translation, one was a public health major fluent in Korean and had lived in the US for over ten years, and the other was a nursing doctor who had lived in the US for over ten years. Finally, the authors compared the reverse-translated instrument with the original English version. All authors confirmed that there were no significant differences in meaning. No further modifications were made except for minor adjustments to improve clarity and correct spelling and grammar.

#### Content validity

To verify the content validity of the Korean version of the RCAC, five oncology nursing experts with at least five years of nursing experience who did not participate in the translation process reviewed the forward–backward-translated instrument. Two of these experts are professors of nursing who specialize in oncology and are actively conducting related research. The other three are working as oncology nurse in hospitals and have master’s degrees in nursing. Their combined expertise ensured a comprehensive evaluation of the instrument’s relevance and accuracy in the context of oncology nursing in Korea. The expert group was asked to evaluate content equivalence using the four-point scale (1 = not relevant, 2 = somewhat relevant but needing modification, 3 = quite relevant but needing some modification, and 4 = highly relevant) presented by Polit et al. [27].

After confirming content validity, the item-level content validity index (I-CVI) and scale-level content validity were calculated by averaging (S-CVI/number of responses). An I-CVI of at least 0.80 and an S-CVI/Ave of at least 0.90 were deemed indicative of adequate content validity [27]. The expert panel confirmed any revisions due to expression, vocabulary, translation clarity, and cultural differences. Based on the opinions of the five experts, the authors engaged in three online discussions and made the necessary corrections.

#### Preliminary survey

A preliminary survey of ten women who met the inclusion criteria based on guidelines was conducted [26]. Participants were given an instrument guide and asked questions about which items were difficult to understand or required modification. The women were asked the following questions: 1) “Was any item hard to understand?”, 2) “Was the content appropriate?”, and 3) “How long did it take to complete the instrument?”.

### Data collection

The data were collected between November and December 2020. Recruitment documents were posted on multiple support group social network services platforms registered on South Korean portal sites. The study details were provided when participants accessed the link. After accessing the website and reviewing the study instructions, participants clicked the “I agree to participate in the study” button to express their readiness to participate. Before the survey, their eligibility was confirmed using an online questionnaire. Participants were asked about their eligibility for the inclusion criteria (e.g., sex, age, diagnosis of cancer, and current treatment) and were asked to withdraw from the survey if they were not eligible. Once eligibility was verified, participants were invited to participate in the study. This was performed to ensure that only patients who met the inclusion criteria were included. Participation was optional, and withdrawal was permitted. Additionally, to prevent multiple participation, the IP addresses and phone numbers collected were checked to provide reimbursement. No duplicate participation was observed.

### Validity test

To verify construct validity, exploratory factor analysis (EFA) was conducted and the criterion validity of the items was verified. EFA was performed with principal component analysis and varimax rotation to extract the latent factors from the data. To evaluate the criterion validity of the Korean version of the RCAC, the FACT-G and PHQ-9 were used. A correlation coefficient less than 0.4 indicates a “weak/very weak or no relationship.” If it is 0.4–0.6, it is deemed “moderate.” If it is 0.6–0.8, it is deemed “strong.” 0.8 or more indicates a “very strong” relationship [28].

### Reliability

The reliability of the newly developed scale was verified using the item-total method. Cronbach’s alpha was used to estimate acceptable internal consistency, which was considered acceptable when > 0.7 [29].

### Data analysis

The IBM SPSS program (version 25.0; IBM Corp., Armonk, NY, USA) was used for statistical analysis. Descriptive statistics were used to summarize the demographic characteristics and item analyses. Construct validity was confirmed using EFA, and Pearson’s correlation coefficients were used to assess criterion validity and reliability. To determine the suitability of the data for factor analysis, the mean, standard deviation, skewness, and kurtosis of each item were analyzed. Additionally, the Kaiser–Meyer–Olkin measure of sampling adequacy and Bartlett's test of sphericity were used [30]. To assess construct validity, EFA was performed using principal component analysis and the Varimax method [31]. An item with a factor loading less than 0.5 was removed in turn, starting with the lowest factor-loading item. The number of factors to be retained was initially determined by cutting off factor eigenvalues of less than one [30, 31]. The retained factors were assessed to ensure that they had at least three items with loadings greater than 0.5, did not cross-load on other factors, and had a total explained variance of at least 50% [31–33].

### Ethical consideration

This study was approved by the Institutional Review Board (IRB No. 202011–0009-01) of the principal investigator’s institution. An online questionnaire was distributed using Google Forms. An information sheet was posted on the front page of the survey to inform participants of the study’s purpose and explain the researchers’ credentials, confidentiality, and the voluntary nature of participation. The participants could view the details of the study, including its purpose, significance, and content, in an online document. Participants could access the questionnaire after providing informed consent by clicking the consent button. In other words, they indicated their willingness to participate before starting the survey. Participation was optional, and participants were allowed to withdraw. Additionally, before starting the survey, questions were created to determine whether the participants met the inclusion and exclusion criteria to ensure that they were suitable candidates for the study.

## Results

### Content validity

The I-CVI and S-CVI/Ave values were calculated as 1.0. Some of the wordings were clarified based on expert advice. Therefore, only a few words were modified and no items were deleted. Specifically, through this discussion, it was necessary to explain the meaning of fertility at the beginning of the survey, rather than in the item of the instrument; therefore, an explanation of the term was added.

### Preliminary survey

There were ten participants in the preliminary survey with an average age of 37 years. The survey participants included four unmarried and six married women. Three women had thyroid cancer, three had breast cancer, three had colon cancer, and one had cervical cancer. In response to the three questions regarding the instrument, three women felt that it might be too difficult for most women to understand. Thus, minor revisions were made to five items (items 2, 3, 5, 6, and 10 from the RCAC) to enhance conciseness and clarity by reordering words and altering expressions. For instance, the item “I am concerned that I may not be able to have (more) children” was translated to “The concern about not being able to have children” in Korean. In item 5, 6, 10 from the RCAC “Having children” is not a frequently used expression in Korea, so it was changed to “become pregnant.” In addition, the wording in item 2 of the RCAC was modified to convey the risk of passing the condition more clearly to a child. Furthermore, to streamline the language and improve clarity for women without a current partner, the phrase ‘spouse or future spouse’ in item 3 of the RCAC was revised to ‘(future) spouse.’ This adjustment emphasizes that ‘future' pertains to potential future circumstances, maintaining the original intent of the instrument.

All ten women agreed that the revised instrument was clear and indicated that the items were appropriate. The mean time required to complete the instrument was 6.5 min. The researchers then finalized the instrument. The average sentence comprehension was 4.28 out of 5 points, and the appropriateness of the item arrangement was 4.18 points.

### Characteristics of the study participants

Table 1 presents the characteristics of the study participants. The average age of the participants was 38.4 (SD = 4.8). The mean age at cancer diagnosis was 35.7 (SD = 5.6) years, and the average time since treatment was completed was 16.6 (SD = 18.8) months. More participants indicated that they did not receive fertility-related treatments, such as fertility preservation, before or after cancer treatment. A higher percentage of participants indicated that they did not desire a child than those who responded “yes” (Table 1).

**Table 1:** Participant characteristics (*N* = 182)

Variable		M ± SD	*n* (%)
Demographic characteristics			
Age (years)		38.38 ± 4.82	
Marriage	Single		29(15.9)
	Married		151(83.0)
	Bereaved & Divorced		2(1.1)
Have a child	Have children		137(75.3)
	No children		45(24.7)
Number of children (*n* = 137)		1.33 ± 0.54	
Education	High school		19(10.4)
	College		152(83.5)
	Graduate school		11(6.0)
Annual household income	High		13(7.1)
	Middle		155(85.2)
	Low		14(7.7)
Occupation status	Unemployed		59(32.4)
	Leave of absence		34(18.7)
	Part-time		36(19.8)
	Full time		49(26.9)
	Student or graduate student		4(2.2)
Cancer characteristics			
Cancer type	Thyroid		59(32.4)
	Breast		52(38.6)
	Ovary		19(10.4)
	Endometrial		12(6.6)
	Cervical		25(13.7)
	Stomach		8(4.4)
	Other		7(3.8)
Age at diagnosis (years)		35.72 ± 5.64	
Stage at diagnosis	0		46(25.3)
	1		102(56.0)
	2		27(14.8)
	3		7(3.8)
Treatment after diagnosis (multiple responses)	Operation		138(37.3)
	Chemotherapy		103(27.8)
	Radiation therapy		91(24.6)
	Targeted therapy		14(3.8)
	Anti-hormonal therapy		22(5.9)
	Other		2(0.5)
Elapsed time after completion of treatment (months)		16.55 ± 18.82	
**Variable**		**M ± SD**	***n*** (%)
Fertility-related experiences			
Fertility treatment experience (before treatment)	No		116(63.8)
	Yes		66(36.3)
Fertility treatment experience (after treatment)	No		106(58.2)
	Yes		76(41.8)
Hope for pregnancy (before treatment)	No		105(57.7)
	Yes		77(42.3)
Hope for pregnancy (after treatment)	No		95(52.2)
	Yes		87(47.8)
Abortion experience	No		102(64.2)
	Yes		57(35.8)

*M* Mean, *SD* Standard deviation

### Exploratory factor analysis

The factor structure of the Korean version of the RCAC was examined using EFA. The Kaiser–Meyer–Olkin score was 0.85, and Bartlett sphericity was χ^2^ = 844.82 (*p* < 0.001), indicating suitability for EFA [30].

When the 18 items in the questionnaire were analyzed in the first EFA, eight items were placed in Factor 1, six in Factor 2, two in Factor 3, and two in Factor 4. When confirming whether each item consistently contributed to the factors’ explanations, out of the 8 items grouped under factor 1, two items (items 12 “I worry that getting pregnant (again) would take too much time and effort.” and 14 “It is stressful to think about trying to get pregnant.”) related to worry and stress related to pregnancy attempts were deleted because they were not consistent with the items that constituted Factor 1. Among the items constituting Factor 3, item 5 “I can accept it if I’m unable to have (more) children.” and item 6 “I am overwhelmed by the thought of trying to get pregnant (again).” were excluded, because they did not indicate the same theme. Item 6, which had a smaller factor loading than item 5, was deleted. A second EFA was performed.

When performing the second EFA, Factor 1 included six items, Factor 2 included six, and Factor 3 included three. The content of the items included in Factor 1 was analyzed and was found to be consistent. Factor 2 was deleted as the factor load of item 11 “Having (more) children will make me more nervous about getting cancer again.” was 0.45 and ≥ 0.50 was required [31–33]. All the items in Factor 3 were retained because the factor loadings and content were appropriate. Finally, the third EFA was performed.

The final Korean version of the RCAC was divided into a total of three factors and 14 items, with a total explanatory power of 57.58% (Table 2). Factor 1 was labeled “fertility potential” with six items and an explanatory power of 33.65%. Factor 2 was named “health problem” with five items and an explanatory power of 14.97%. Factor 3 was named “acceptance” with three items and an explanatory power of 8.95%.

**Table 2:** Exploratory factor analysis for the Korean version of the RCAC (*N* = 182)

Korean version sub-scale	Content	M ± SD	Factor 1	Factor 2	Factor 3
Fertility potential	I am afraid that I won’t be able to have any (more) children	2.65 ± .96	**0.74**	0.18	0.03
	I am worried about my ability to get pregnant (again)		**0.76**	0.18	0.15
	I am concerned that I may not be able to have (more) children		**0.77**	0.22	0.05
	I worry about telling my (potential) spouse/partner that I may be unable to have children		**0.75**	0.16	0.14
	I am concerned that my (potential) spouse/partner will be disappointed if I can’t get pregnant		**0.73**	0.10	0.17
	The thought of telling my (potential) spouse/partner that I may be unable to have children makes me uncomfortable		**0.69**	0.13	0.07
Health problems	I am worried about passing on a genetic risk for cancer to my children	3.17 ± .85	0.18	**0.73**	-0.06
	I am worried about how my family history might affect my children’s health		0.01	**0.77**	0.19
	I am afraid my children will have a high risk of cancer		0.15	**0.76**	0.02
	I am scared of not being around to take care of my children someday		0.17	**0.70**	0.00
	I am cautious about having (more) children because I might not be around to raise them		0.24	**0.66**	-0.10
Acceptance	I can accept it if I’m unable to have (more) children	2.68 ± .86	0.03	-0.25	**0.64**
	I will be happy with life whether or not I have (more) children		0.09	0.15	**0.76**
	I will feel content if I do not have (more) children		0.35	0.05	**0.67**
	Eigen-values		4.71	2.10	1.25
	Explanatory power (%)		33.66	14.97	8.95
	Cumulative variance (%)		33.66	48.63	57.58

*RCAC* Reproductive Concerns After Cancer Scale, *M* Mean, *SD* Standard deviation

### Criterion validity

We confirmed criterion validity by analyzing the correlation between the Korean version of the RCAC and both the FACT-G and PHQ-9 (Table 3). The FACT-G and PHQ-9 demonstrated significant but moderately low correlation coefficients with the Korean version of the RCAC (*r* = -0.36 (*p* < 0.001) and *r* = 0.38 (*p* < 0.001), respectively).

**Table 3:** Correlation between Korean version of the RCAC and FACT-G and PHQ-9 (*N* = 182)

Factor	Korean version of the RCAC				FACT-G	PHQ-9
	**Factor 1**	**Factor 2**	**Factor 3**	**Total**		
Factor 1 fertility potential	1					
Factor 2 health problem	0.40^***^	1				
Factor 3 acceptance	0.31^***^	0.02	1			
Total Korean version of the RCAC	0.88^***^	0.73^***^	0.46^***^	1		
FACT-G	-0.36^***^	-0.24^**^	-0.10	-0.36^***^	1	
PHQ-9	0.43^***^	0.18^*^	0.12	0.38^***^	-0.68^***^	1

*RCAC* Reproductive Concerns After Cancer Scale, *FACT-G* the Functional Assessment of Chronic Illness Therapy-General scale, *PHQ-9* Patient Health Questionnaire-9

^*^p < .05

^**^p < .01

^***^p < .001

### Reliability

Cronbach's alpha coefficient of the 14 items of the Korean version of the RCAC was 0.83. The Cronbach’s alpha coefficients of the “fertility potential,” “health problem,” and “acceptance” subscales were 0.79, 0.80, and 0.82, respectively.

## Discussion

Appropriate oncofertility care improves the quality of life in cancer survivors. Nurses are responsible for the fertility of cancer survivors while delivering care [34]. They require instruments to assess and communicate with cancer survivors and healthcare providers. However, a suitable instrument to measure reproductive concerns among cancer survivors is not yet available in South Korea. We translated the RCAC and evaluated its psychometric properties in a Korean context. This study is significant because it provides evidence supporting the use of the Korean version of the RCAC as a reliable instrument for assessing reproductive concerns in effective oncology care.

An EFA and criterion validity analysis were conducted to confirm the construct validity of the Korean version of the RCAC. Based on the EFA results, we revised the factor structure, collapsing and deleting items to better align with prevailing cultural perceptions in Korea. These perceptions emphasize family stability and group identity, which significantly influence views on health and fertility. The Korean version of RCAC consists of three subscales — “fertility potential,” “health problems,” and “acceptance” — comprising 14 items in total. Each subscale was adapted to reflect the specific cultural context of Korea, distinct from the original RCAC and other international versions. This adaptation emphasizes the need for culturally specific modifications to ensure the instrument’s relevance and effectiveness.

The original RCAC published in English [6, 15] and other Chinese [17] and Swedish [2] versions had six subscales, while the Portuguese version [18] had five subscales. There are some differences in the factor names across countries. The six original American subscales were as follows: 1) fertility potential, 2) partner disclosure, 3) child’s health, 4) personal health, 5) acceptance of possible infertility, and 6) becoming pregnant. In the Korean version of the RCAC, the subscales include “fertility potential,” “health problems,” and “acceptance.” Specifically, fertility potential and partner disclosure were combined into one factor, “fertility potential.” The second factor consisting of child and personal health was combined into “health problems.” Finally, the last factor, "acceptance", remained unchanged. Pregnant women (items 6, 12, and 14) were eliminated from the list. This unique factor structure may be influenced by two factors. The first concerns the cultural characteristics of South Korea and Asia. In South Korea, women prioritize family stability and collective identity [19, 35]. As the primary caregivers for 91.3% of children in 2018 [36], they often considered their children as extensions of themselves [19, 20]. In this cultural context, women may perceive a close connection between their health and their children’s well-being. This characteristic appears to have led to the perception of partners and children as “my family” and can be seen as a cause of changes in subscales such as partner disclosure, child health, and personal health. Second, some modifications were made because of the low awareness and understanding of oncofertility among patients and clinicians in South Korea compared with other countries [37]. The differences in the Chinese version’s [17] subscales are attributed to varied infertility perceptions between China and Korea despite their shared Asian regions. Based on a systematic review of the reproductive concerns experienced by cancer survivors [38], South Korea may be less familiar with oncofertility than China.

During the psychometric evaluation, four items (6, 11, 12, and 14) were eliminated. Items 6, 12, and 14 related to pregnancy and future pregnancies were removed. It is believed that the deletion of the factor of becoming pregnant was due to cultural differences and varying perceptions of oncofertility. In Korea, it has been noted that cancer survivors abandon their desire to become pregnant [37]. Additionally, previous studies have shown a lack of active discussion regarding the fertility of cancer survivors [5, 37]. These cultural environments may have contributed to the elimination of these factors.

Item 11 refers to concerns regarding cancer recurrence due to future pregnancies. As indicated above, the higher average age of our participants compared with other studies likely resulted in them not considering the possibility of future pregnancies. Consequently, they may perceive pregnancy-related health issues as irrelevant to their circumstances. The birth rate is increasing among Korean women aged > 35 years old. The average age for first-time mothers is 33.0 years in Korea, which is the highest among OECD countries [9, 10]. Consequently, compared to previous studies [6, 15, 17, 18], our results may have been influenced by our population’s higher maternal age and the fact that 75.3% of the participants already had children.

Pregnancy and fertility issues are largely overlooked among Korean cancer survivors. For "acceptance", which is composed of the same items as the original instrument, our results were similar to those of the original version [6] because the items concerned being able to accept the difficulties of future pregnancy. Similarly, for Items 2, 4, 9, and 18, which reflect the present circumstances with children, the results were similar to those of the original version. These items were retained in the questionnaire. Specific items regarding future pregnancies may have been influenced by a lack of awareness about oncofertility. Previous studies have shown that Korean women are unfamiliar with pregnancies after cancer [37]. Specifically, female cancer survivors in South Korea face unexpected effects on their fertility due to cancer treatment and often struggle with a lack of information until after treatment. In this study, approximately 60% of the participants reported that they did not receive fertility-related treatments, such as fertility preservation, before or after cancer treatment, suggesting that both clinicians and patients are unaware of fertility issues after cancer care. 

We confirmed criterion validity by correlating the quality of life (FACT-G) and depressive symptoms (PHQ-9) with the Korean version of the RCAC. The Korean version of the RCAC was negatively correlated with the FACT-G and positively correlated with the PHQ-9, indicating criterion validity, which is in line with previous research [5, 6]. Because the FACT-G and PHQ-9 do not measure the same construct as the Korean version of the RCAC but are related to the Korean version of the RCAC, significant, moderately low correlations are evidence of criterion validity. Cronbach’s alpha of the Korean version of the instrument ranged from 0.79 to 0.82, depending on the factor, and the overall reliability of the instrument was 0.83, which was similar to or slightly higher than that of the original English version [6] and other versions [17, 39]. Therefore, the Korean version of the RCAC is a reliable and valid instrument for assessing reproductive concerns.

In addition, 47.8% of the participants expressed a desire to become pregnant after completing cancer treatment. Our results aligned with previous studies indicating that issues related to fertility can persist over a cancer survivor’s lifetime [2, 3, 36, 40]. Even five years after treatment, cancer survivors experience physical and psychosocial difficulties related to fertility [40]. Nurses can use the Korean version of the RCAC to assess cancer survivors’ reproductive concerns and provide appropriate care.

To our knowledge, this is the first study to determine the validity and reliability of an instrument measuring reproductive concerns among female cancer survivors in South Korea. These results will help to clarify and assess the reproductive concerns of young Korean cancer survivors. This may support the development of guidelines or policies to provide care for those with reproductive concerns who require nursing care.

### Limitations

This study has several limitations. First, most participants reported that cancers occur in the thyroid and breast, and future studies should include a wider variety of cancer types. Second, as this study focused on verifying the instrument’s reliability and validity, it did not examine differences in reproductive concerns among those who underwent fertility treatments, including fertility preservation, before and after treatment. Future studies should be conducted on Korean cancer survivors to investigate these differences. Third, these results were generated using only EFA, without Confirmatory Factor Analysis for cross-validation. Therefore, additional studies should ascertain whether the Korean version of the RCAC remains a valid and reliable instrument for assessing reproductive concerns after conducting a Confirmatory Factor Analysis. Fourth, a significant strength of this study was its online data collection method, which allowed the recruitment of a diverse pool of participants. However, the absence of subsequent test–retest evaluations may be considered a limitation of this study. Fifth, most participants were in their mid-30s, with a smaller proportion being single or without children. Future research should target younger women, including teenagers and young adults, and aim to include single women and those without children. Furthermore, future studies should identify differences in RCAC based on population characteristics, such as age (teenagers and young adults), presence of children, and current pregnancy status. Finally, the developed instrument was shorter than the original instrument when certain items were eliminated, and some factors were integrated during psychometric testing. The Korean version of the instrument may have been improved by the incorporation of other instruments of reproductive concern.

## Conclusions

This study aimed to develop and validate a Korean version of the RCAC to assess the reproductive concerns of female cancer survivors. The validity and reliability of the instrument were confirmed for measuring reproductive concerns after cancer treatment in Korean women. The Korean version of the RCAC consists of the following three factors with 14 items: 1) fertility potential, 2) health problems, and 3) acceptance. The modified factors in the Korean version of the RCAC were influenced by Korean characteristics, including culture, high maternal age, and low awareness of oncofertility. This instrument can be used to assess and counsel cancer survivors regarding their reproductive health. This study underscores the need for healthcare professionals, particularly nurses, to use such instruments to understand and address the reproductive concerns of cancer survivors in their care. The Korean version of the RCAC can inform the development of guidelines and policies that go beyond identifying the reproductive health needs of cancer survivors to provide tailored interventions. Future research should be conducted on younger, unmarried women and those without children.

## Data Availability

The participants did not provide written informed consent for their data to be shared publicly. Owing to the sensitive nature of the research, the supporting data are not available.

## Abbreviations

RCAC: Reproductive Concerns After Cancer Scale
FACT-G: Functional Assessment of the Cancer Therapy-General Scale
PHQ-9: Patient Health Questionnaire-9
I-CVI: Item-level content validity index
S-CVI/Ave: Scale-level content validity by averaging
EFA: Exploratory factor analysis
SD: Standard deviation
